# Stratospheric influence on surface ozone pollution in China

**DOI:** 10.1038/s41467-024-48406-x

**Published:** 2024-05-14

**Authors:** Zhixiong Chen, Jane Liu, Xiushu Qie, Xugeng Cheng, Mengmiao Yang, Lei Shu, Zhou Zang

**Affiliations:** 1 Key Laboratory for Humid Subtropical Eco-Geographical Processes of the Ministry of Education, School of Geographical Sciences, Fujian Normal University, Fuzhou, China; 2 Institute of Atmospheric Physics, Chinese Academy of Sciences, Beijing, China; 3 Department of Geography and Planning, University of Toronto, Toronto, ON Canada

**Keywords:** Atmospheric chemistry, Environmental impact

## Abstract

Events of stratospheric intrusions to the surface (SITS) can lead to severe ozone (O_3_) pollution. Still, to what extent SITS events impact surface O_3_ on a national scale over years remains a long-lasting question, mainly due to difficulty of resolving three key SITS metrics: frequency, duration and intensity. Here, we identify 27,616 SITS events over China during 2015-2022 based on spatiotemporally dense surface measurements of O_3_ and carbon monoxide, two effective indicators of SITS. An overview of the three metrics is presented, illustrating large influences of SITS on surface O_3_ in China. We find that SITS events occur preferentially in high-elevation regions, while those in plain regions are more intense. SITS enhances surface O_3_ by 20 ppbv on average, contributing to 30-45% of O_3_ during SITS periods. Nationally, SITS-induced O_3_ peaks in spring and autumn, while over 70% of SITS events during the warm months exacerbate O_3_ pollution. Over 2015-2022, SITS-induced O_3_ shows a declining trend. Our observation-based results can have implications for O_3_ mitigation policies in short and long terms.

## Abstract (web summary)

The authors analyze the frequency, duration and intensity of stratospheric intrusions to the surface in China over 2015-2022 and find that such intrusions enhance surface ozone pollution, especially in spring and autumn, followed by summer.

## Introduction

Compelling evidence has suggested that high ozone (O_3_) concentrations in the surface layer can be harmful to human health and vegetation growth^1^. As known, tropospheric O_3_ originates from two sources: photochemical production within the troposphere and dynamical injection from the stratosphere. Though the injected stratospheric O_3_ is estimated to account for only 5-10% of the tropospheric O_3_ sources^2^, case studies of stratospheric intrusions (SI) to the troposphere have documented how SI occurred and impacted tropospheric and even surface O_3_^3–5^. Events of deep and fast stratospheric intrusions to the surface (SITS) can lead to high-O_3_ episodes, inducing severe O_3_ pollution^6–9^. In the recent decade, China has confronted a severe O_3_ pollution problem, even after the implementation of strict policies on emission reductions of O_3_ precursors. The causes for this environmental issue have been investigated from the perspectives of chemical responses to the changes in both emissions and meteorology^10–12^. Yet, the contribution of natural stratospheric O_3_ inputs to surface O_3_ pollution is often neglected. Verstraeten et al.^13^ found a substantial positive trend in stratospheric contributions to tropospheric O_3_ over China for 2005-2010 based on numerical simulations. It is still highly uncertain about the influence of SITS on the surface O_3_ over a long period. Such long-term variation of stratospheric influence on surface O_3_ is not only an issue for China, but also for elsewhere in the world^3^.

Though SITS events are transient and limited in local areas, their impact on surface O_3_ can be substantial during SITS periods over the affected areas, regarding the absolute O_3_ enhancement and fractional contribution to overall surface O_3_. For example, Chen et al.^14^ showed that a SITS event during the COVID-19 lockdown period in 2020 enhanced surface O_3_ concentrations in Beijing, China, by 8 ppbv, which contributed to 23% of overall surface O_3_. These extra stratospheric O_3_ inputs, plus the background O_3_, can exacerbate air pollution beyond the recommended O_3_ threshold. The SITS-induced O_3_ exceedances depend not only on the absolute SITS O_3_ inputs, but also on surface background O_3_, which is modulated by the interplay of multiple chemical and physical processes, varying at different time scales, and sensitive to O_3_ precursor emissions and environmental conditions. Therefore, to what extent SITS events are harmful to human health and crop yield is largely uncertain, not only in their absolute magnitudes, but also in relative contributions to surface O_3_. To our knowledge, this issue has not been resolved on a national scale for the worsening ground-level O_3_ pollution in China to date.

Assessing the impact of SITS on surface O_3_ for large areas over long periods requires a good understanding of three key SITS metrics: frequency, duration, and intensity. These three metrics are essential to evaluate the O_3_ exposure associated with SITS concerning the health effect. Despite the knowledge obtained from SITS case studies, it is challenging to obtain an overview of SITS events in terms of these three metrics on a national, continent, or global scale, due to the difficulty of explicitly resolving them. For stratospheric air to reach the surface, it has to be transported downward across the first barrier, the tropopause, through deep SI, and then quickly descends and crosses the second barrier, the planetary boundary layer (PBL)^15–17^. Insufficient consideration of multi-scale atmospheric dynamical processes, including large-scale tropopause folding and small-scale PBL mixing, can easily lead to biased representation of the frequency, duration, and intensity of SITS events. Chemical transport models are often applied to evaluate stratospheric O_3_ transported into the troposphere using tagged stratospheric tracers^18,19^, but the estimated stratospheric influences are heavily dependent on tracer definitions and resolvable dynamical processes^20,21^. Especially, the abundant and complicated chemical sink compounds of O_3_ in the PBL would result in enhanced uncertainty of the intruded stratospheric O_3_ if they are not accurately described in models^7,16^. The instantaneous and local nature of SITS events^3^ requires comprehensive observations with a high spatial and temporal resolution in order to assess their impacts on surface O_3_. The routine weekly ozonesonde observations can directly monitor vertical O_3_ variation, they are not able to capture SITS events that persist only for several hours^4,8,9^. Running multi-instrumental campaigns with targeted stratospheric tracers, such as O_3_ and cosmogenic radionuclide, can provide reliable indicators of SITS^5,22,23^. Performed in limited regions and periods, however, these campaigns are spatially and temporally too scant to provide a national-scale estimation of stratospheric influences on surface O_3_ for long periods. Therefore, how can we overcome our insufficient ability to achieve a nation-wide assessment of SITS events regarding their frequency, duration, and intensity?

Stratospheric air is characterized by high O_3_, low carbon monoxide (CO), and low relative humidity (RH)^3,4,24^. Intrusions of stratospheric air downward to the surface level produce sharp upward (downward) spikes in the surface-measured O_3_ (CO), as documented in many case studies^6–9^. Such stratospheric signals can provide a clear indication of SI that have reached the surface level with the aid of dense surface observations. China has built a nationwide network consisting of more than 1600 surface stations that are capable of providing hourly observations of O_3_, CO, NO_2_ and SO_2_ concentrations^25,26^. This comprehensive dataset is both spatially and temporally dense, so it is possible that the data from this network could capture the signals of SITS events over large areas much more effectively than those mentioned traditional data, and hence provide an opportunity to investigate the stratospheric contribution to surface O_3_ and its long-term trends across the nation.

Here, we take advantage of the dense surface observations and develop a SITS detection method using O_3_ and CO as stratospheric indicators and their distinct spikes during SITS as constraints (see “Methods” section), based on our previous case studies^9,14^. This method bypasses the need for detailed knowledge about the multi-scale dynamical processes and complicated chemical sinks for the descending stratospheric air. The general features of SITS from our detection method are in line with those derived from multi-instrumental observations in published literatures (see ”Methods” section). This gives us confidence to resolve the spatial and temporal variations of SITS events in China. Basing on large samples of SITS events detected locally at individual stations across the nation in 8 years (27,616 events in total), here we aim to address the following scientific questions: (1) what are the characteristics of SITS events in China over 2015-2022 in terms of frequency, duration, and intensity? (2) to what extent SITS-induced O_3_ impacts surface O_3_ variations and contributes to the occurrences of surface O_3_ pollution? and (3) what is the trend of SITS-induced O_3_ in China over 2015-2022?

## Results

### Spatial distributions and seasonal variations of SITS in China

**Fig. 1:**
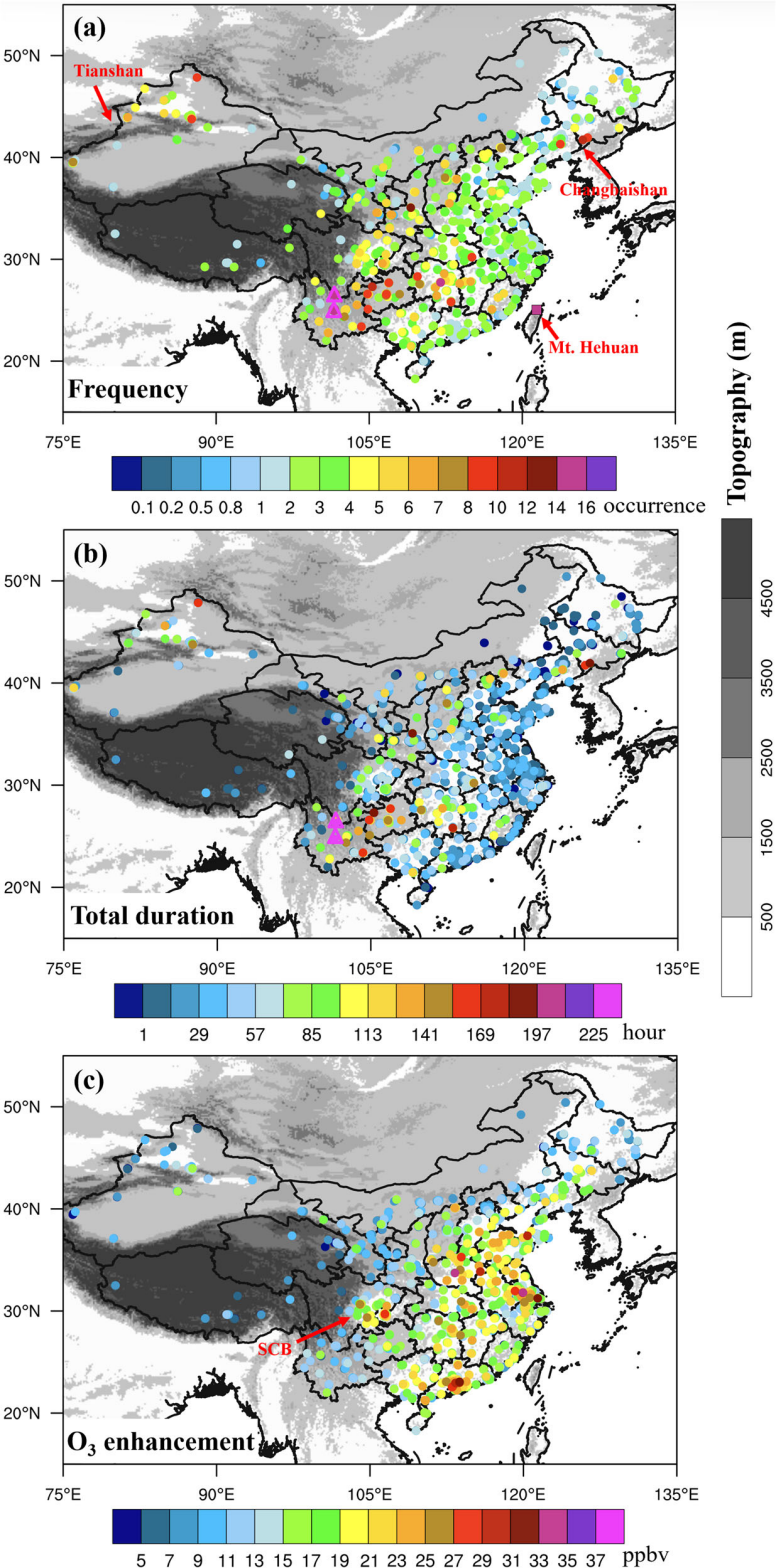
Spatial distributions of the three key metrics of stratospheric intrusions to the surface (SITS) events in China. **a** The annual frequency (unit: occurrence per year), (**b**) the annual total duration (unit: hours per year) and (**c**) the surface O_3_ enhancements (unit: ppbv) of SITS events averaged over 2015-2022. Mt. Changbaishan, Tianshan and Hehuan are indicated in (**a**), and locations of Panzhihua and Chuxiong are marked by the magenta triangles. SCB in (**c**) refers to the Sichuan Basin. Source data are provided as a Source Data file.

A total of 27,616 SITS events were screened out during 2015-2022 using surface observations from 1500 stations with continuous 8-year measurements in China (see “Methods” section). Figure 1 shows the spatial distributions of the annual frequency, annual total duration, and surface O_3_ enhancements of the SITS averaged over 2015-2022. The hot spots for SITS frequency and duration are located in high-elevation regions in the southeast rim of the Tibetan Plateau in southwest China, Tianshan Mountains in northwest China and Changbai Mountains in northeast China (Fig. 1a, b). The annual SITS frequency averaged is 8-12 occurrences per year over these regions, with an average of the total duration of 120-150 h per year, i.e., ~1.7% of the entire year. The highest SITS frequency is 16 and 14 occurrences per year in cities Panzhihua (3130 m above sea level) and Chuxiong (2530 m above sea level), both of which are situated closely to the Tibetan Plateau. Correspondingly, the total duration of SITS there reaches 345 hours and 318 hours per year, respectively, accounting for 3.94% and 3.63% of the time in a year. In the plain areas of eastern China, however, SITS events are relatively sparse and rare with a mean frequency of 2–3 occurrences per year and a total duration of 30-60 hours annually.

Figure 1c shows the SITS-induced O_3_ enhancements above their corresponding reference baselines averaged over the SITS duration (see “Methods” section for the baseline definition). Unlike the spatial variations in frequency and duration, the surface O_3_ enhancements are larger in plain than in high-elevation areas. In central and eastern China, average surface O_3_ enhancements are 15–25 ppbv during the SITS, while in western China, where elevations are high, only 7–15 ppbv. The SITS-induced O_3_ enhancements varying with elevation are more evident in the Sichuan basin (SCB). The reasons for such O_3_ enhancements varying with elevation can be subject to further studies. Here we suggest that for those SITS events descending to plain regions, the air parcels originated in the stratosphere have to travel a deeper vertical extension experiencing more chance to be diluted by tropospheric air. Hence, only those strong SITS events are more likely to reach the surface over plain regions. Trickl et al.^27^ suggested that the intensity of O3 enhancements during the SITS partially depend on how high the intrusions start in the stratosphere. Possibly, SITS events over plain regions in eastern China may initiate at higher altitudes within the stratosphere.

**Fig. 2:**
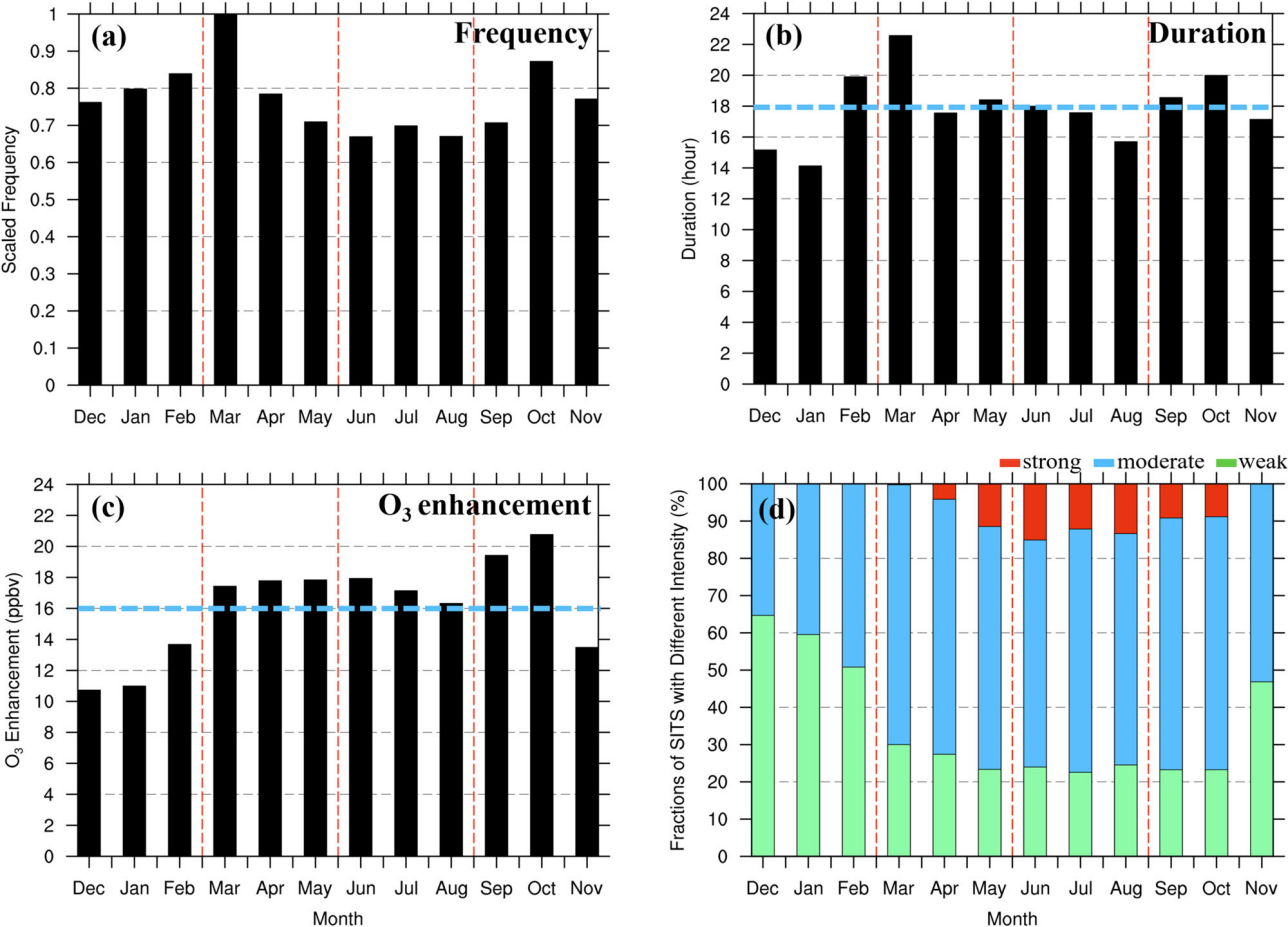
Monthly variations in the three key metrics of stratospheric intrusions to the surface (SITS) events in China. **a** Frequency of SITS events scaled to the maximum value in March. **b** Duration of STIS events in each month (unit: hours). **c** O_3_ enhancements of SITS events relative to the corresponding surface O_3_ reference baselines (unit: ppbv). The horizontal blue dashed lines in **b** and **c** represent the corresponding annual means. **d** Fractions of SITS associated with different intensity categories, i.e., weak (O_3_ enhancements<15 ppbv, green bar), moderate (15 ppbv<O_3_ enhancements<40 ppbv, blue bar), and strong (O_3_ enhancements >40 ppbv, red bar). The four seasons are separated by red dashed lines for winter (December-February), spring (March-May), summer (June-August), and autumn (September-November). Source data are provided as a Source Data file.

The frequency of the SITS exhibits distinct seasonality with a maximum in early spring, a secondary maximum in autumn and a minimum in summer (Fig. 2a). Such seasonality is in agreement with multiple studies of SI climatology in the midlatitudes of the Northern Hemisphere^28–32^. In particular, the number of detected SITS events in China shows a pronounced peak in March and a minimum in August. In terms of the duration of the SITS events, responding to the rapid mixing processes with tropospheric air and abundant chemical sinks, SITS-induced O_3_ can elevate surface O_3_ concentrations above the baseline for 18 hours on average (Fig. 2b). In general, SITS occurring in spring and autumn persist longer than in winter and summer. Besides showing the highest SITS frequency, March also exhibits the longest duration of SITS events, yielding elongated stratospheric impacts on surface O_3_. Note that the duration here does not include the time before which stratospheric O_3_ is chemically destroyed in the troposphere, instead, it refers to the period when the stratospheric air parcel reaches the surface and retains its rich-O_3_ and poor-CO properties that are distinguishable from tropospheric air.

The SITS-induced O_3_ enhancements averaged over the SITS duration in each month are compared in Fig. 2c. Generally, the stratospheric O_3_ enhancements are stronger in warmer months, with an average of 18.1 ppbv above the baseline value from March to October, and weaker during colder months, with an average of 12.2 ppbv above the baseline. Furthermore, Fig. 2d illustrates the distinct seasonal cycles of the SITS events with three levels of intensity measured by O_3_ enhancements^27^. The SITS events with O_3_ enhancements exceeding the surface O_3_ baseline by less than 15 ppbv (weak SITS) exhibit a maximum in cold months, while those with an exceedance over 40 ppbv (strong SITS) appear frequently in warm months, especially in summer. The distinct seasonality of SITS with different intensities is in line with Trickl et al.^27^, who attributed the maximum of stratospheric O_3_ intensity in summer to the air parcels’ higher origins in the stratosphere than in the other seasons. Due to an elevated tropopause in summer^33^, the summertime SITS events originating at higher altitudes would have high O_3_ concentrations because the altitudes are closer to the stratospheric O_3_ reservoir ~20–25 km.

### Substantial stratospheric contribution to surface ozone pollution episodes

**Fig. 3:**
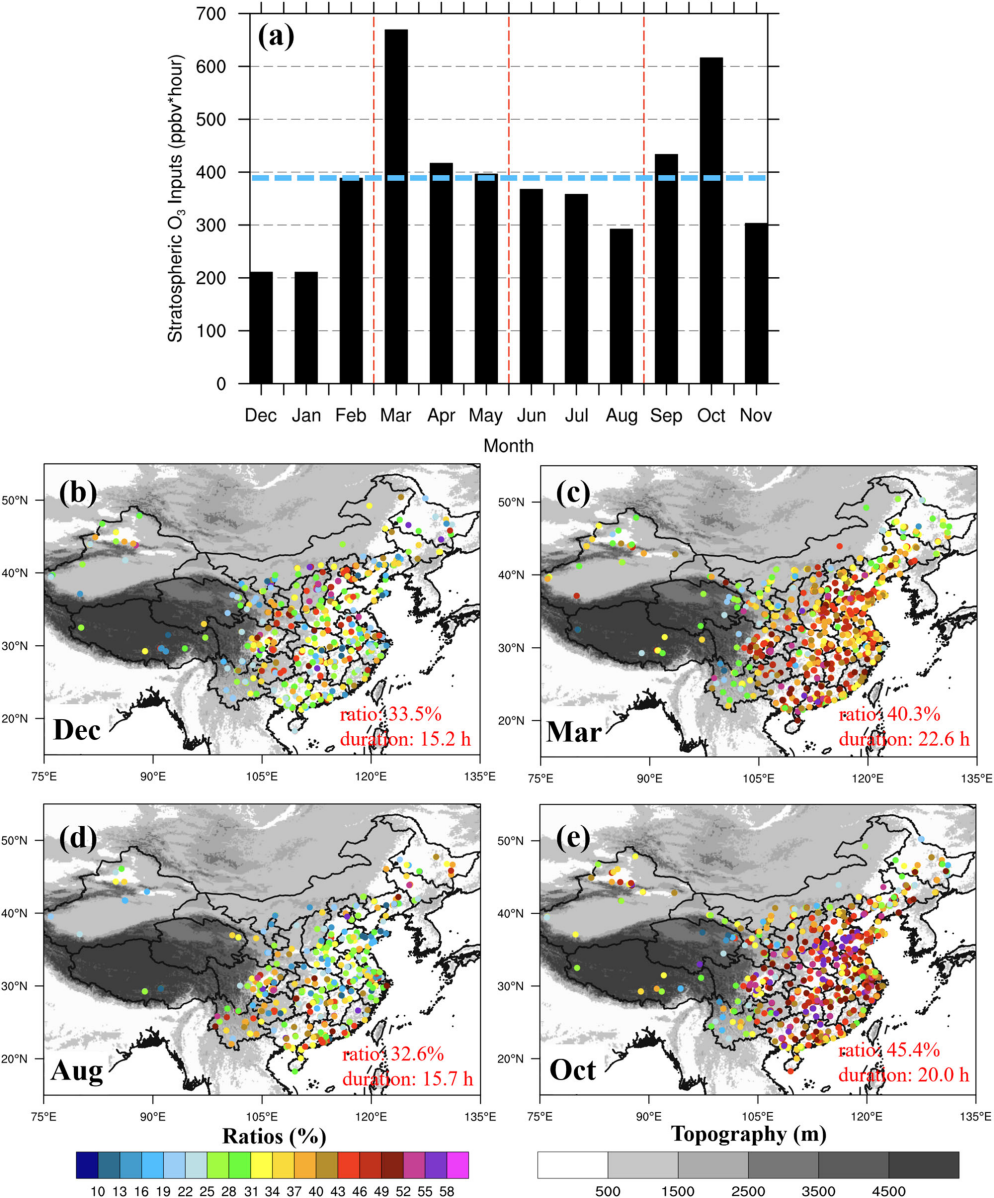
The monthly sum of stratospheric O_**3**_ inputs (${{{{{{\boldsymbol{O}}}}}}}_{{{{{{\bf{3}}}}}}}^{{{{{{\boldsymbol{strat}}}}}}}$) and the ratios of ${{{{{{\boldsymbol{O}}}}}}}_{{{{{{\bf{3}}}}}}}^{{{{{{\boldsymbol{strat}}}}}}}$ to the sum of surface O_3_ concentrations during stratospheric intrusions to the surface (SITS) events (${{{{{{\boldsymbol{Ratio}}}}}}}_{{{{{{\boldsymbol{SITS}}}}}}}$). **a** The monthly sum of ${O}_{3}^{{strat}}$ (unit: ppbv*hour; see Eq. (5) in “Methods” section) in China averaged over 2015-2022. The horizontal blue dashed line represents its annual mean. **b**–**e** Spatial distributions of ${{Ratio}}_{{SITS}}$ during the periods of the SITS events (unit: %; see Eq. (4) in “Methods” section) in December, March, August and October. The red numbers in the lower right corner are the mean ${{Ratio}}_{{SITS}}$ and the mean duration of SITS events in the corresponding months on a national scale. Source data are provided as a Source Data file.

Based on a complete depiction of the three key SITS metrics, we estimate the magnitude of injected stratospheric O_3_ at ground level by integrating the hourly excess of O_3_ concentrations relative to their reference baselines^28^ (Fig. 3a, unit: ppbv*hour, see “Methods” section). Averaged over 2015-2022, the monthly stratospheric O_3_ inputs to the surface layer in China show a peak in March, a second peak in October and a minimum in December and January. Governed by the three key metrics of SITS, it is clear that stratospheric influences maximize in early spring due to the high frequency and duration and moderate intrusion intensity. The combination of high intrusion intensity and moderate frequency and duration of SITS in autumn leads to a second peak of stratospheric influences. The intrusion frequency is relatively high in winter (Fig. 2a); however, the short duration and weak magnitudes of the SITS (Fig. 2b, c) result in the least stratospheric inputs to surface O_3_ in winter. During the short periods of the SITS (referred to as the SITS duration), these additional stratospheric O_3_ inputs substantially enhance the surface O_3_ concentrations, consisting of 30-45% of surface O_3_ over SITS-affected areas (Fig. 3b–e). The ratio of stratospheric O_3_ to overall surface O_3_ can reach 58% in March and October, calling for extra consideration of the stratospheric influence on O_3_ budgets in the 2 months.

**Fig. 4:**
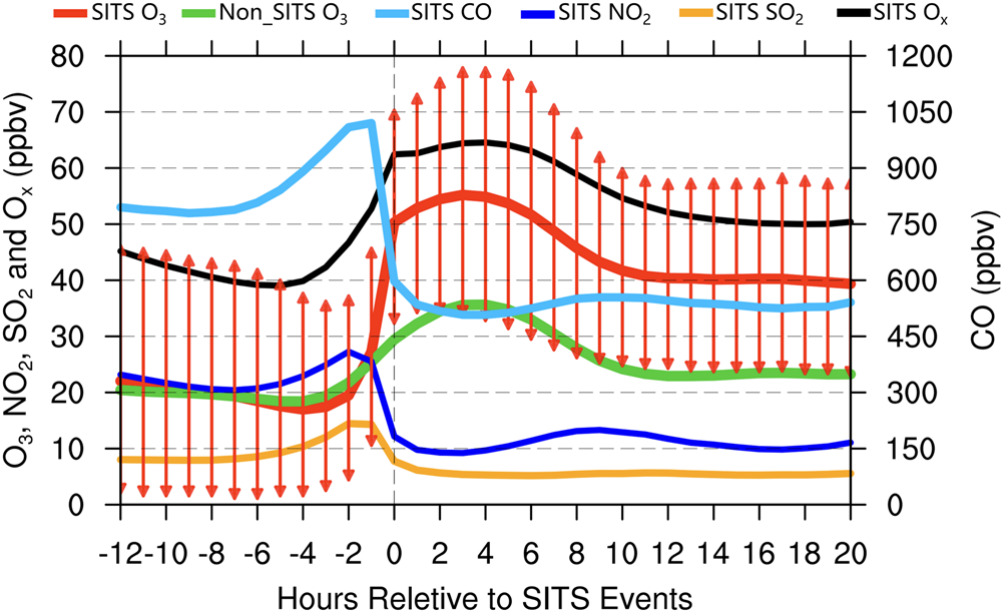
Composite analysis of hourly O_3_, CO, NO_2_, SO_2_ and total oxidants (O_x_ = O_3_ + NO_2_) concentrations during all detected stratospheric intrusions to the surface (SITS) events in China over 2015-2022. The zero hour represents the start hour of all SITS events. A period of 33 h is applied starting 12 h before the SITS start hour and ending 20 h after the start hour. O_3_ concentrations of each SITS events (SITS O_3_, red solid line, unit: ppbv) are aligned into the 33-h period and averaged in each hour. The red arrows measure the 10th and 90th percentile of SITS O_3_ concentrations. The same procedure is applied to the hourly O_3_ reference baselines (Non_SITS O_3_, green solid line), CO (SITS CO, light blue solid line), NO_2_ (SITS NO_2_, deep blue solid line), SO_2_ (SITS SO_2_, orange solid line), and O_x_ (SITS O_x_, black solid line). Source data are provided as a Source Data file.

The natural stratosphere-to-troposphere (STT) processes have direct influences on the chemical compositions in the troposphere^34^. Figure 4 presents a statistical overview of the instantaneous impacts of SITS on surface gaseous compounds, from 12 h before the start of the SITS to 20 hours after, averaged over all the detected SITS events. The synchronously sharp enhancement of O_3_ and reduction of CO, the two indicators of SITS used in our detection method, are obvious at the moment when SITS events start. Compared with the baseline values representing the non-SITS conditions, surface O_3_ is generally enhanced by 20 ppbv in the initial hours when stratospheric O_3_ reaches the surface and still retains its stratospheric properties largely, leading to a positive O_3_ anomaly of 60–70%. Right after SITS occurrences, the 90th percentile of surface O_3_ concentrations is directly enhanced by up to 40 ppbv above their normal values, which can affect adversely human health and ecosystems.

**Fig. 5:**
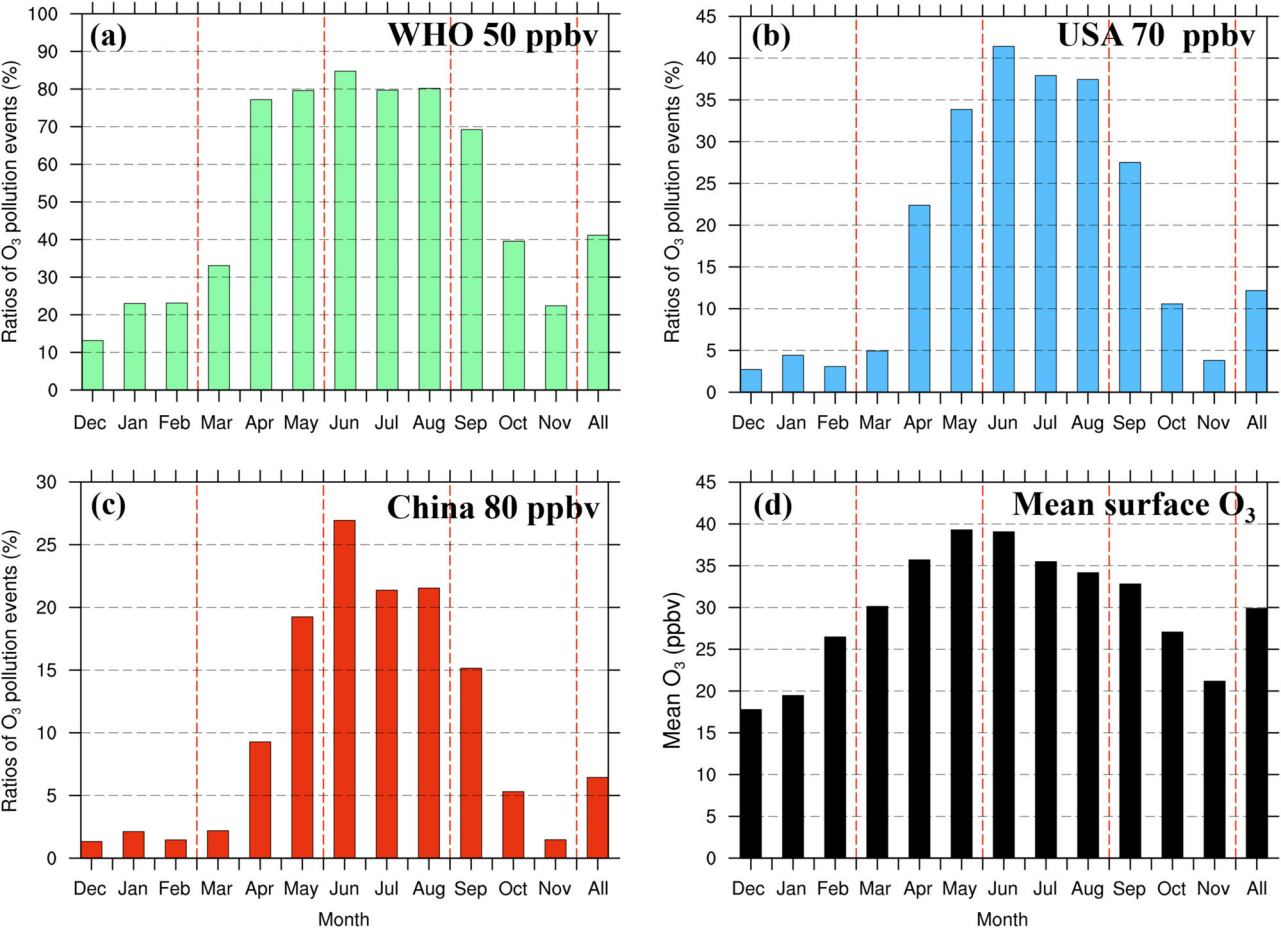
The fraction of stratospheric intrusions to the surface (SITS) events with O_3_ exceedance to the total SITS events (unit: %) under different air quality standards and mean surface O_3_ concentrations in each month over 2015–2022. The recommended O_3_ standard by the World Health Organization (WHO) is 50 ppbv (**a**) measured as 8-h maximum moving average within a day (MDA8), while it is 70 ppbv (**b**) and 80 ppbv (**c**) in the USA and China, respectively. In each detected SITS event, the MDA8 O_3_ is calculated and compared against the standards above to determine the occurrence of O_3_ exceedance. **d** Mean surface O_3_ concentrations (unit: ppbv) averaged over all surface stations in each month_._ The monthly variations in the fraction and surface O_3_ concentrations are the mean over 2015–2022, while the rightmost column represents the annual mean. Source data are provided as a Source Data file.

The recommended O_3_ threshold by the World Health Organization (WHO) is 50 ppbv^35^, which is measured as a daily maximum 8-h average (MDA8), while the threshold is 70 ppbv in USA^7^, and 80 ppbv in China^12^. When MDA8 O_3_ is larger than the threshold, O_3_ exceedances occur. Figure 5 shows the fraction of SITS events with O_3_ exceedances (based on the three O_3_ thresholds above) to the total number of SITS events in each month, averaged over all the stations and all the years. Here for each standard, if there is at least one O_3_ exceedance during a SITS event, we regard the event as a SITS-induced O_3_ exceedance. Referring to the WHO standard (Fig. 5a), over 70% of SITS events are associated with O_3_ exceedances from the middle of spring to summer, with an annual mean ratio of 41.1%. Similarly, the inputs of stratospheric O_3_ can lead to high O_3_ exposure to more harmful-level concentrations, even above the US and Chinese O_3_ standards (Fig. 5b, c). In spring, O_3_ in the lowermost stratosphere builds up and frequent tropopause folding events take place^36–38^. In addition, the seasonal variation in the surface background O_3_ shows an overall maximum in spring and summer in China^39^ (Fig. 5d). Under such conditions, air pollution is greatly exacerbated by SITS events from April to September, raising great health concerns especially during the warm months.

For CO variations during SITS periods as shown in Fig. 4, the composite analysis indicates a reduction of 30-35% compared with their values before SITS occurrences. Other gas pollutants that are not used for identifying SITS events here, such as NO_2_ and SO_2_, also exhibit a prominent decline during the SITS hours. We also examined the responses of atmospheric oxidation capacity (AOC) to SITS using total oxidant (O_x_=O_3_ + NO_2_) as an indicator. Despite a reduction of NO_2_ when SITS events occur, the injected stratospheric O_3_ compensates for the loss of NO_2_ and instantly increases the AOC by 20-35%. As a consequence, the enhanced AOC would help stimulate the formation of particulate nitrate and secondary organic aerosols^40^. Stratospheric air injected into the surface can substantially alter the tropospheric air compositions, reaction sensitivity regimes between O_3_-NO_x_-VOC and tropospheric oxidative states^34,41,42^. Even though SITS events are transient, the complicated perturbation from the stratosphere induces changes in the balanced tropospheric air and can amplify the initial effects of SI^18,43–45^. Therefore, SITS should be seriously considered in tropospheric chemistry and air pollution control^46^.

### Declining influences of SITS on surface ozone variations in 2015-2022

**Fig. 6:**
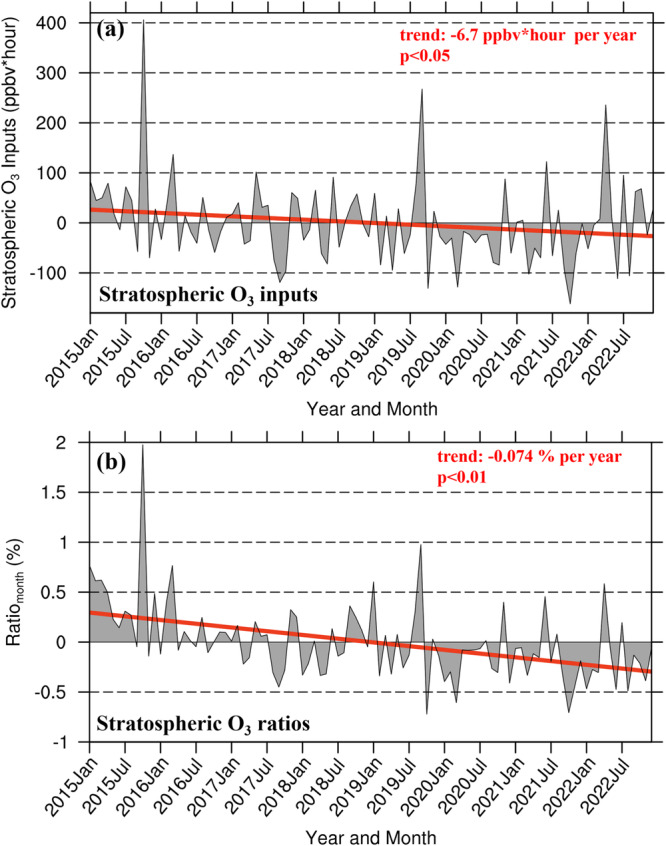
Deseasonalized monthly stratospheric O_3_ inputs and their ratios to surface O_3_ concentrations averaged at all study sites in China over 2015-2022. The gray shaded areas represent the time series of deseasonalized monthly means of (**a**) stratospheric O_3_ inputs (see Eq. (5) in “Methods” section) and (**b**) their ratios to the surface O_3_ concentrations (see Eq. (6) in “Methods” section). The red lines represent the linear trends for stratospheric O_3_ inputs and stratospheric O_3_ ratios, respectively, and the red numbers in the upper right corner are the trends that are statistically significant above the 95% and 99% confidence level, respectively. Source data are provided as a Source Data file.

The quasi-decadal observations enable us to examine the long-term variation of stratospheric influence on surface O_3_. Supplementary Fig. 1a shows variations in the monthly stratospheric O_3_ inputs to the surface (in the unit of ppbv*hour, see “Methods” section, Eq. (5)) in China over 2015-2022 and their ratio to the overall surface O_3_ during SITS periods in the affected areas (see “Methods” section, Eq. (4)). The ratio ranges between 25-50%, and shows substantial interannual variations. Over 2015-2022, the magnitude of direct stratospheric influence on surface O_3_ across China appears declining. Figure 6a further shows the time series of deseasonalized monthly accumulated SITS-induced O_3_ over 2015-2022. A declining trend significantly at a 95% level is observed at a rate of -6.7 ppbv*hour per year, which is ~1–2% per year. An independent indicator of the stratospheric influence, the occurrence of very-high-O_3_ concentrations during SITS (e.g., 80–100 ppbv; Supplementary Fig. 2), also exhibits a decreasing trend, supporting the results of Fig. 6a.

If scaled to longer timescales of a month or year (see Eq. (6) in “Methods” section), the ratio of stratospheric inputs to surface O_3_ is much lower (Fig. 6b and Supplementary Fig. 1b). On a annual basis, 1.6-2.2% of surface O_3_ is attributable to the stratospheric inputs in the affected areas. The stratospheric O_3_ ratios at the surface exhibit a maximum in early spring (2.7%) and autumn (2.2%), but a minimum in the summer (1.3%). Low fractions of stratospheric O_3_ inputs to surface O_3_ over annual or longer periods have been documented. For example, Cristofanelli et al.^47^ estimated that deep SI contributed 2% of surface O_3_ on the southern slope of the Himalayan region, and Lin et al.^48^ found that the SI contributed 1.3% of surface O_3_ in Mt. Hehuan of Taiwan.

Over 2015–2022, the ratio of SITS-induced O_3_ to overall surface O_3_ concentrations also declined significantly at -0.074% per year (Fig. 6b). The model simulations of Verstraeten et al.^13^ suggested increasing stratospheric contributions to the tropospheric O_3_ increases in China over 2005–2010. Yet, based on the analysis of surface observations, this study suggests a minor and declining stratospheric influence on the surface O_3_ increase in China over 2015–2022, at least for the direct and deep SI events. The causes for the declining trend of stratospheric influences may include the weakened O_3_ abundance in lower stratosphere^49,50^ (Supplementary Fig. 3a) where STT events take place mostly, and the reduced strong SITS occurrences (Supplementary Fig. 2). Regarding the tropospheric environments where stratospheric O_3_ intruded into, the capping stable layer (thermal inversion) tends to descent and intensify since 2010 in China (Supplementary Fig. 3b), hindering the formation of deep PBL for downward transport of stratospheric air to the surface^15,16^. The stratospheric O_3_ injected to the troposphere averaged over China, deriving from the Trajectory-mapped Ozonesonde dataset for the Stratosphere and Troposphere (TOST)^51^ data, also indicates a declining tendency over 2015-2022 (Supplementary Fig. 4). Supplementary Fig. 5 presents the estimated amounts of stratospheric O_3_ in the surface layer from the MERRA-2 GMI global atmospheric chemistry model^52^, which also shows a decreasing trend of stratospheric contributions to surface O_3_.

## Discussion

Estimates of the stratospheric influences on surface O_3_ are necessary for making effective mitigation policies, since these inputs of natural stratospheric O_3_ can substantially enhance the risk of O_3_ pollution episodes and partially determine the floor value for air quality managements. In a short term, SITS-induced O_3_ has non-negligible significance for transient high-O_3_ episodes, given its large fractions of surface O_3_ budget (30-45%) and high risks of O_3_ exposure to harmful-level concentrations during SITS periods in affected areas. While the absolute stratospheric influences are highest in March and October, special attention to O_3_ pollution control should be paid in spring and summer when extra SITS-induced O_3_ inputs, plus the high background O_3_, promote possibility to exacerbate O_3_ pollution beyond the WHO and national standards. In 10% of the SITS cases, surface O_3_ can be elevated by over 40 ppbv, setting alarms for possible severe O_3_ pollution in affected areas. On the other hand, SITS could synchronously reduce concentrations of other air pollutants, including CO, NO_2_, and SO_2_. Such stratospheric perturbation can also substantially enhance the oxidation capacity of tropospheric air. On the annual basis, detectable O_3_ with stratospheric origins consists of 1.6–2.2% of surface O_3_ in China, implying that O_3_ pollution mitigation over long terms in China should mainly focus on surface O_3_ variations through photochemical reactions under the influence of meteorology and anthropogenic emissions. In this study, we conservatively estimate the stratospheric influences by only including the dynamically injected stratospheric O_3_ associated with deep, direct, and fast SITS events. The influences of aged stratospheric air injected into the troposphere from the stratosphere are spared, since such influences could not be easily identified from surface measurements. A combination of satellite remote sensing technology and deep machine learning methods in future work can help solve these issues. The chemically-induced O_3_ production due to stratospheric perturbation may also contribute to surface O_3_ variations in the presence of nonlinear chemical reactions during SITS^53,54^. The above factors can amplify the influences of the stratosphere especially in transient surface O_3_ pollution events, and hence enhance the impact of O_3_ on human health and crop yield.

## Methods

### Screening SITS based on comprehensive surface observations

Taking advantage of surface gaseous pollutant measurements, e.g., O_3_, CO, NO_2_, and SO_2_, with a high spatial and temporal resolution, here we develop a methodology of detecting SITS events over large areas and for long periods, based on and further refined from our previous studies^9,14^. This method is effective in detecting deep, direct, and fast SI retaining stratospheric properties, such as “O_3_-rich and CO-poor”^3,4,15^. In this study, we focus on such SITS events, while aged stratospheric air that has reached the surface but lost its stratospheric properties is not considered. Relying on the characteristics of stratospheric air reaching the surface (richer O_3_ and poorer CO relative to tropospheric air), we identify a SITS event based on hourly concurrent O_3_ and CO measurements at the surface based on the following points.

1. Distinct upward and downward spikes of O_3_ and CO indicators, respectively. The hourly measurements of stratospheric indicators (O_3_ and CO) are screened to filter out their distinct spikes, e.g., the sharp increase in O_3_ and decrease in CO simultaneously, a unique indication for air with recent stratospheric origins. Hourly variations of O_3_ and CO concentrations throughout the year are calculated site by site and year by year. The 95th percentile of the O_3_ rising rate and 5th percentile of the CO decline rate in each year are chosen to identify those sudden and sharp spikes when stratospheric air initially reaches the surface^55^. The synchronous appearance of extreme O_3_ increase and CO decrease could help isolate the sudden surface O_3_ change due to SI from that due to O_3_ transport or photochemical processes. As shown in Supplementary Fig. 6, the two parameters simultaneously determine the start timing of a SITS event (SITS_start).
2. The large departures of O_3_ and CO from their normal values. The intruded stratospheric air contains higher O_3_ than that in the troposphere; therefore, surface O_3_ with additional inputs in SITS events is supposed to increase from its normal values. To minimize the blurring of photochemically produced tropospheric O_3_, we consider that the O_3_ concentrations at the SITS_start hour should exceed the seasonal mean value during noontime (${\bar{{O}_{3}}}^{{noon}}$, 1st O_3_ criterion) when photochemical reactions are active. Simultaneously, the CO concentrations during the SITS should decline below their seasonal mean value (CO criterion). These criteria could also help remove the occasions that could be falsely identified when O_3_ is transported downward from the residual layer, an O_3_-rich “reservoir” containing photochemically produced O_3_ in the preceding day^56,57^. Due to the mixing with tropospheric air and chemical sinks of O_3_, the properties of the intruded stratospheric air subside over the time^58^. When the O_3_ concentrations fall back to their seasonal mean values (${\bar{{O}_{3}}}^{{season}}$, 2nd O_3_ criterion) or the CO concentrations rebound over the CO criterion, stratospheric air is not distinguishable from the tropospheric air and hence the SITS events end (SITS_end; referred to the case illustrated in Supplementary Fig. 6).

Provided with the start and end timing of SITS events, we estimate the amounts of injected stratospheric O_3_ reaching the surface by integrating the excess of O_3_ concentrations above their reference baselines (the seasonal means at the corresponding hour) during the SITS events (see details in the next section). At a given station for a period, such as a month, the number of SITS occurrences, the length of time between SITS_start and SITS_end averaged over all SITS events in the period, and the averaged excess of O_3_ concentrations above the baselines are regarded as the frequency, duration, and intensity of the SITS at that station for that period.

Similar to the definition of a chemical tropopause^59^, we rely on the variations in atmospheric chemical constituents O_3_ and CO, rather than some dynamic indicators, to define the frequency, duration, and intensity of SITS events. These definitions are referred throughout this manuscript. Both O_3_ abundance in the lower stratosphere and frequency of deep stratosphere-to-troposphere processes primarily determine the injected amounts of stratospheric O_3_ into the troposphere^60^. When intruded into the troposphere, stratospheric O_3_ can be strongly mixed with tropospheric air and be chemically destroyed, responding to the complicated dynamical and chemical processes in the troposphere, especially in the PBL. Therefore, assessing the stratospheric contribution to surface O_3_ depends on not only the detailed information of SITS (e.g., their frequencies and magnitudes), but also the varying tropospheric environments that control the fate of injected stratospheric O_3_ (SITS duration).

Although stratospheric air is also characterized with low RH, RH is not selected as an indicator in our detect algorithm. This is because RH is inversely related to temperature. Low RH may also appear when air parcels descend from higher altitudes to the lower troposphere experiencing adiabatic warming. The air parcels can also experience various atmospheric moisture conditions on their way to the surface, so RH of the air parcels is less conservative than O_3_ and CO^3,8^. In addition, concurrent RH measurements are usually unavailable in air quality monitoring stations in China.

We have developed this SITS methodology with a goal of being objective, robust, and accurate, i.e., reducing the commission and omission errors as much as possible. We have inclined to be conservative and set the detecting criteria rather strictly. For example, we assure that SITS would enhance surface O_3_ concentrations, i.e., as long as surface O_3_ concentrations are not above the background value, SITS stops. In this way, the detected SITS events are highly likely to be the cases, while some weak SITS events may be omitted.

### Estimation of contributions of SITS to surface O_3_

The contributions of SITS to surface O_3_ are estimated event by event and station by station. The hourly mean surface O_3_ concentrations (${\bar{O}}_{3}^{h}$; where h = 1, 2, 3,…24) are calculated by averaging O_3_ observations at each of the 24 hours in each season based on station-level observations in each year. The ${\bar{O}}_{3}^{h}$ values are taken as reference baselines to measure the departure of O_3_ concentrations from their baselines during SITS periods. Provided with the start and end timing of SITS events, we integrate the excess of O_3_ above the reference baselines during SITS periods (unit: ppbv*hour), and take it as the amount of injected stratospheric O_3_ (${O}_{3}^{{strat}}$) in each SITS event^14,28,31,48^:

1 $${O}_{3}^{{strat}}={{\int }}_{{SITS}{{\_}}{start}}^{{{SITS}{{\_}}{end}}} ({O}_{3}^{h}-\,{\bar{O}}_{3}^{h}){dt}$$

where ${O}_{3}^{h}$ denotes the in situ hourly O_3_ observations at hour *h*, and the ${\bar{O}}_{3}^{h}$ represents the baseline O_3_ concentrations at the same hour. The differences between ${O}_{3}^{h}$ and ${\bar{O}}_{3}^{h}$ are summed over the SITS period with a temporal resolution of 1 hour (i.e., d*t* = 1 hour).

The sum of O_3_ concentrations during each SITS event and its corresponding month (unit: ppbv*hour) are calculated following Eqs. (2) and (3), respectively:

2 $${{O}_{3}}_{{SITS}}^{{sum}}={{\int }}_{{SITS}{{\_}}{start}}^{{{SITS}{{\_}}{end}}}{O}_{3}^{h}{dt}$$

3 $${{O}_{3}}_{{month}}^{{sum}}={{\int }}_{{month}{{\_}}{start}}^{{{month}{{\_}}{end}}} {O}_{3}^{h}{dt}$$

The ratio of stratospheric O_3_ (${O}_{3}^{{strat}}$) to the sum of O_3_ concentrations during each SITS event (${{Ratio}}_{{SITS}}$) is given by Eq. (4):

4 $$R{{atio}}_{{SITS}}=\frac{{O}_{3}^{{strat}}}{{{O}_{3}}_{{SITS}}^{{sum}}} * 100\%$$

The sum of stratospheric O_3_ inputs during all SITS events in a month (${{O}_{3}}_{{month}}^{{strat}}$, unit: ppbv*hour) is calculated by Eq. (5), given the number of SITS events in the month being *N*:

5 $${{O}_{3}}_{{month}}^{{strat}}={\sum }_{i=1}^{N}{O}_{3,i}^{{strat}}$$

Finally, the ratio of stratospheric O_3_ to the sum of O_3_ concentrations in the corresponding month (${{Ratio}}_{{month}}$) is given by Eq. (6):

6 $$R{{atio}}_{{month}}=\frac{{{O}_{3}}_{{month}}^{{strat}}}{{{O}_{3}}_{{month}}^{{sum}}} * 100\%$$

The time series (2015–2022) of SITS-induced O_3_ and its ratio to overall surface O_3_ concentrations during SITS periods and the entire month are showed in Supplementary Fig. 1.

### Validations of the SITS detection algorithm

As SITS appears as rare events in local areas, it is important to verify the reliability of our detection algorithm in order to address the impact of deep SI on surface O_3_. We previously published a detailed analysis of two SITS events that occurred in China, which were selected from the large samples of SITS events^9,14^. The stratospheric origins and transport pathways of the two SITS cases were revealed by means of surface air pollutant observations, vertical profiles of RH and O_3_, PV evolution, and backward trajectory simulations. The general characteristics of SITS detected with our algorithm, such as their seasonality and contribution to surface O_3_, are in good agreement with the observed deep SI climatology by Stohl et al.^24^, Elbern et al.^28^ and Cristofanelli et al.^31^. The detected frequency of SITS is further compared with published observational studies. For example, based on multiple stratospheric tracers including RH, CO, and cosmogenic radionuclide ^7^Be, Lin et al.^48^ identified 14 SI days during a 13-month campaign in a high-elevation station (3380 m asl) located in Mt. Hehuan of Taiwan (Fig. 1a). The annual frequencies of SITS in Panzhihua (16 per year) and Chuxiong (14 per year) detected in the present study agree reasonably with those at Mt. Hehuan which is with a similar latitudes and altitudes (Fig. 1a). Using a combination of stratospheric tracers including RH, potential vorticity (PV), ^7^Be and the tropopause height anomaly, Cristofanelli et al.^31^ reported that there were 33 days (average 5.5 days per year) of deep and direct SI which were characterized by distinct stratospheric properties at Mt. Cimone (2165 m asl) in Italy over 1998-2003. We apply the SITS detection algorithm to the O_3_ and CO measurements collected in Mt. Cimone during 2013–2016, and find a total of 26 direct SI events (average 6.5 days per year). The results from our detection algorithm are in line with these SI studies shown above and indicate the feasibility of using sudden and sharp spikes of O_3_ and CO to identify SI reaching the surface.

The origins of SITS events are investigated with backward trajectory simulations of 10 days over selected cities (Supplementary Fig. 7; see details of the backward trajectory simulations in the following section). We select Panzhihua as an example where the highest SITS frequency is detected. The cities Beijing and Fuzhou are also selected to examine SITS occurred in northern and southern China, respectively. The trajectory analysis indicates that the majority of air parcels at the surface during the detected SITS events originated in the upper troposphere and lower stratosphere (UTLS; above 400 hPa), i.e., 96% in Panzhihua, and 100% in Fuzhou and Beijing. As another piece of evidence, the ensemble RH profiles during the 33 SITS events over Beijing show substantial dryness characterized by RH < 30% in the PBL and near the ground level^15^, suggesting the dry stratospheric air has descended into the surface. In addition to these selected cities, we further analyze the ensemble of backward trajectories associated with the detected 27,616 SITS events (Supplementary Fig. 8). We evenly divide every trajectory from the beginning to the end into three travel stages. The height, PV, and O_3_ concentrations in each stage are extracted from MERRA-2 reanalysis data. The air parcels of SITS initially reside in 300-200 hPa, where PV values exceed 2 PVU (an iso-surface representing the dynamical tropopause) and O_3_ concentrations are larger than 250 ppbv^61,62^, showing prominent stratospheric origins.

### Backward trajectory simulations and MERRA-2 reanalysis data

Backward trajectories are simulated to check the origins of airmass of detected SITS events (Supplementary Fig. 7 and Supplementary Fig. 8) using the Hybrid Single-Particle Lagrangian Integrated Trajectory (HYSPLIT) model. HYSPLIT is developed by National Oceanic Atmospheric Administration’s (NOAA)^63^ (https://www.arl.noaa.gov/hysplit). The 10-day backward trajectories are driven by the meteorological data from the Global Forecast System (GFS) with a resolution of 0.25°. The PV values and O_3_ concentrations along the trajectory are extracted from the Modern-Era Retrospective analysis for Research and Applications version 2 (MERRA-2) reanalysis data. The MERRA-2^64^ reanalysis data have a spatial resolution of 0.5° latitude × 0.625° longitude with 72 model levels (https://gmao.gsfc.nasa.gov/reanalysis/MERRA-2; DOI: 10.5067/WWQSXQ8IVFW8).

### Surface observational data and stratospheric O_3_ tracer data

The present study is mainly based on analysis of hourly ground-based measurements of O_3_, CO, SO_2_, and NO_2_ concentrations at more than 1,600 stations in Chinese cities. For O_3_ and CO, they are measured with a CO analyzer (Thermo Fisher Model 48i) and an O3 analyzer (Thermo Fisher Model 49i). The detection limit (precision) for Model 48i and Model 49i are 0.04 ppmv (±0.1 ppmv) and 0.5 ppbv (±1 ppbv), respectively. The data are from the public website of the Chinese Ministry of Ecology and Environment (MEE) (https://english.mee.gov.cn/).

To explore the variations in stratospheric O_3_ during the study period (Supplementary Fig. 3a), the stratospheric O_3_ profile observations are acquired from the Stratospheric Water and OzOne Satellite Homogenized (SWOOSH) dataset^65^ (https://csl.noaa.gov/groups/csl8/swoosh). SWOOSH provides a merged record of stratospheric O_3_ on the basis of a number of limb sounding and solar occultation satellites from 1984 to the present.

To investigate the thermal inversion variations over China during 2015-2022 (Supplementary Fig. 3b), radiosonde observations in China are processed, which are available from https://www.ncei.noaa.gov/products/weather-balloon/ integrated-global-radiosonde-archive.

The Trajectory-mapped Ozonesonde dataset for the Stratosphere and Troposphere (TOST)^49,66^ is a 3-dimensional O_3_ dataset derived from ozonesondes at over 100 stations using a trajectory-based mapping methodology with the HYSPLIT model. The thermal tropopause height is determined for each O_3_ profile, and the stratospheric O_3_ distribution is mapped for the O_3_ with stratospheric origination along the trajectory paths. All O_3_ values along the trajectory paths are binned into grids of 5° × 5° × 1 km (latitude, longitude, and altitude) from sea level to 26 km in each month. TOST has been validated against independent ozonesondes and widely used in global O_3_ climatology studies^67^. In the present study, we further extend the TOST data by conducting 10-day forward trajectories simulations over 2015-2021 (Supplementary Fig. 4).

Simulations from the MERRA-2 GMI global chemical transport model are analyzed for long-term variations in stratospheric O_3_ inputs to the surface-layer during the period (Supplementary Fig. 5). The MERRA-2 GMI (Global Modeling Initiative’s) model with stratosphere-troposphere chemical mechanisms is driven by MERRA-2 meteorology including winds, temperature and pressure^52^ (https://acd-ext.gsfc.nasa.gov/Projects/GEOSCCM/MERRA2GMI/). This model is run at a MERRA-2 native horizontal resolution of ∼50 km with 72 vertical levels. The model applies a stratospheric O_3_ tracer to diagnose the stratospheric O_3_ influence on the troposphere.

### Supplementary information

Supplementary Information

Peer Review File

### Source data

Source Data

Featured Image Suggestion

## Data Availability

The datasets used in this study are freely available and are available from the corresponding authors on request. All data supporting the findings of this study are available within the paper and are provided as a Source Data file. Source data are provided with this paper.

## Code availability

All of the figures are created by the authors using the NCAR Command Language Version 6.5.0 (available at http://www.ncl.ucar.edu/). The codes used in this study are available from the corresponding authors on request.
