# Supplementary material for: Stratospheric influence on surface ozone pollution in China
Source: Nat Commun. 2024 May 14;15:4064. doi: 10.1038/s41467-024-48406-x (PMC11093980; doi:10.1038/s41467-024-48406-x)
Supplement: Supplementary file 1 — Supplementary Information [file 41467_2024_48406_MOESM1_ESM.pdf]

Supplementary information for

Stratospheric influence on surface ozone pollution in China

Zhixiong Chen[1,2], Jane Liu[1,3*], Xiushu Qie[2*], Xugeng Cheng[1], Mengmiao Yang[1], Lei Shu[1], Zhou Zang[3]

[1]Key Laboratory for Humid Subtropical Eco-Geographical Processes of the Ministry of Education, School of Geographical Sciences, Fujian Normal University, Fuzhou, China

[2]Institute of Atmospheric Physics, Chinese Academy of Sciences, Beijing, China

[3]Department of Geography and Planning, University of Toronto, Toronto, Ontario, Canada

Correspondence to: Jane Liu (janejj.liu@utoronto.ca) and Xiushu Qie (qiex@mail.iap.ac.cn)

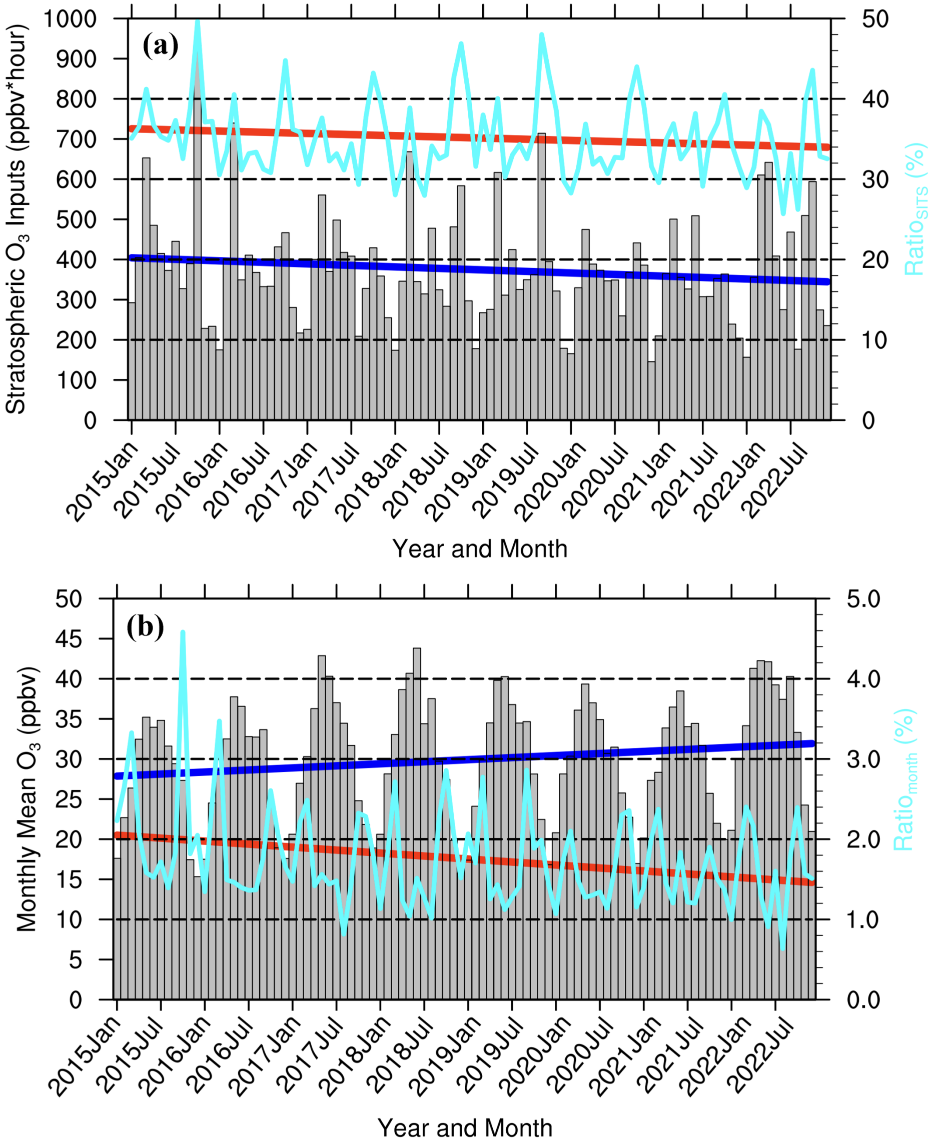

Supplementary Figure 1. Monthly stratospheric O_3 inputs and ratios of stratospheric O_3 to overall surface O_3 concentrations in China over 2015-2022. (a) The excess of O_3 during each stratospheric intrusions to the surface (SITS) event relative to the reference baselines are integrated (O_3^{strat}) in each month (grey bars, unit: ppbv*hour, see Eq. (5) in Methods) with a linear regression fit (blue line). The ratios of O_3^{strat} to the overall surface O_3 concentrations during short periods of SITS events ($Ratio_{SITS}$, unit: %, see Eq. (4) in Methods) and their linear regression fit are shown in the cyan line and red line, respectively. (b) Monthly mean surface O_3 concentrations averaged over all surface stations (grey bars, unit: ppbv) and their linear regression fit (blue line). The ratios of O_3^{strat} to the overall surface O_3 concentrations in each month ($Ratio_{month}$, unit: %, see Eq. (6) in Methods) and their linear regression fit are shown by the cyan line and red line, respectively.

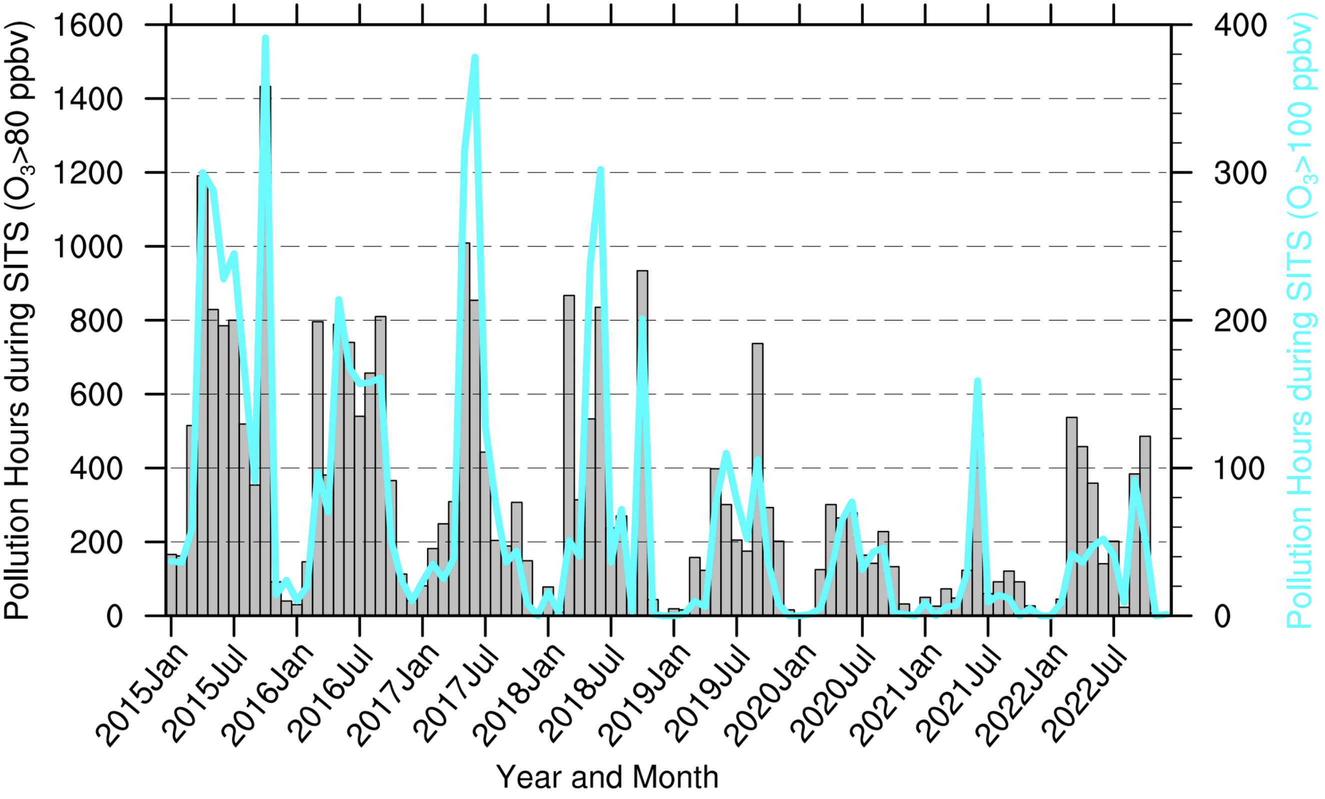

Supplementary Figure 2. Monthly accumulated O_3 pollution hours during stratospheric intrusions to the surface (SITS) events. The sum of hours with O_3 concentrations exceeding 80 ppbv (grey bars) and 100 ppbv (cyan line) during all SITS events in China over 2015-2022.

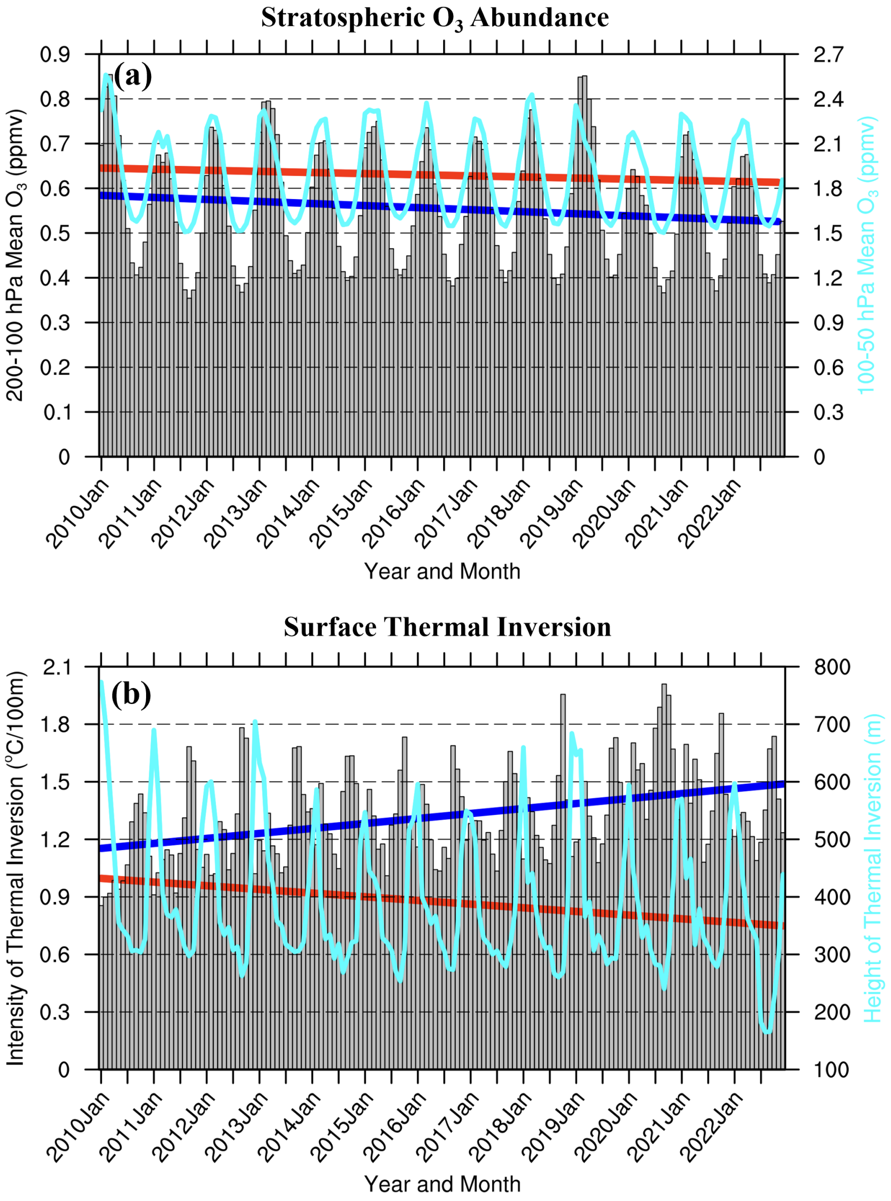

Supplementary Figure 3. Temporal evolution of stratospheric O_3 abundance and surface thermal inversion over 2010-2022. (a) The stratospheric O_3 concentrations (unit: ppmv) from SWOOSH data averaged over 200-100 hPa (grey bars) and 100-50 hPa (cyan line) in lower stratosphere averaged over 30 °N-70 °N. The linear trends for stratospheric O_3 in 200-100 hPa and 100-50 hPa are fitted by the blue and red straight lines, respectively. (b) The height of thermal inversion above the surface (ΔH; cyan line; unit: m) and intensity of thermal inversion (grey bars; unit: °C $100m^{-1}$) based on radiosonde observations over China. The intensity of thermal inversion represents the difference between the warmest temperature above the surface and the surface temperature (ΔT) divided by the ΔH. The linear trends for the intensity and height of thermal inversion are shown by the blue and red straight lines, respectively.

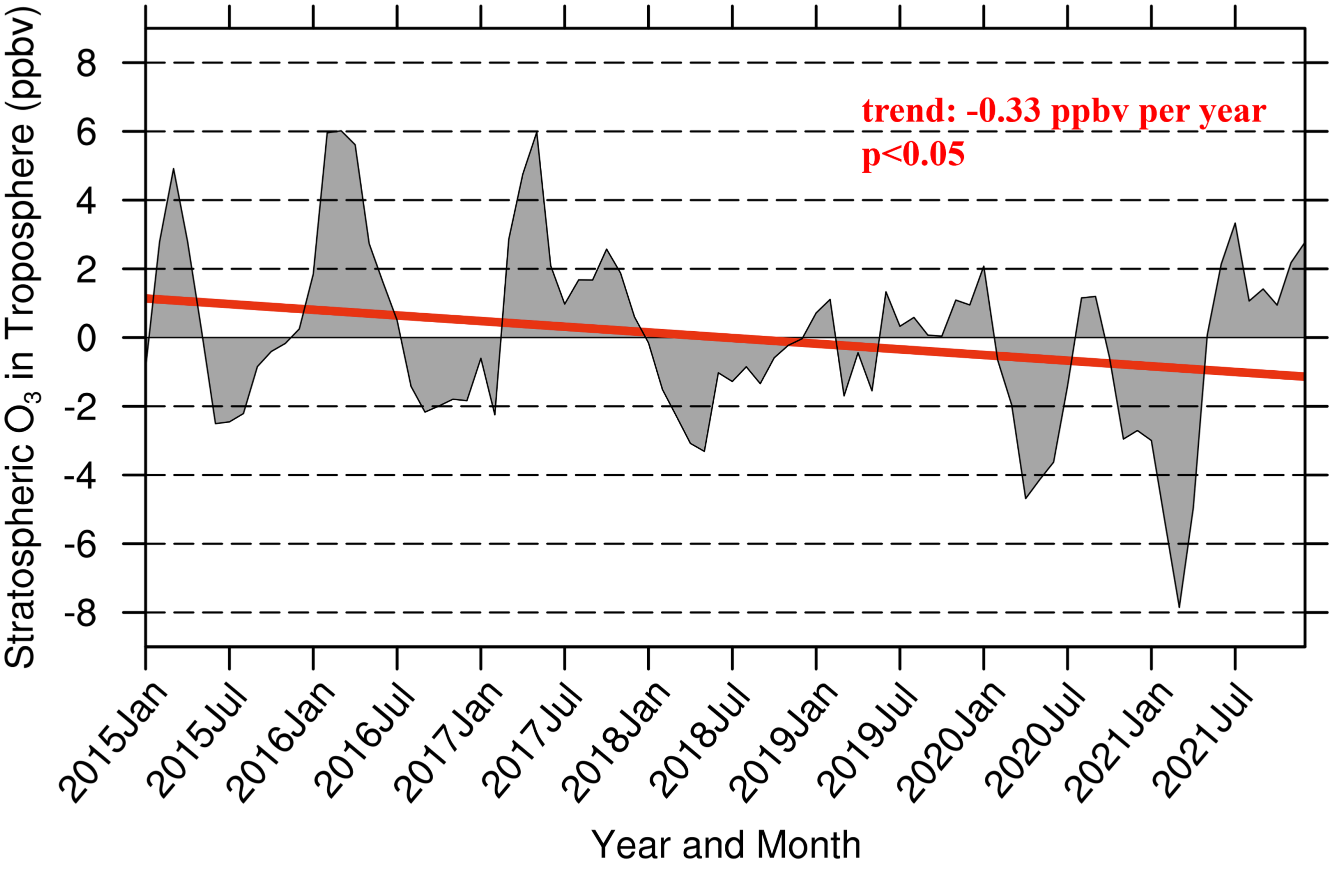

Supplementary Figure 4. Deseasonalized monthly mean stratospheric O_3 injected into troposphere (0-10 km) from TOST data in China over 2015-2021. Values of stratospheric O_3 concentrations injected into troposphere are smoothed using a 3-monthly running mean, and the grey shaded areas represent the time series of deseasonalized monthly stratospheric O_3 concentrations. The red line represents the linear trend with a decreasing rate of -0.33 ppbv per year, which is statistically significant at the 95% confidence level.

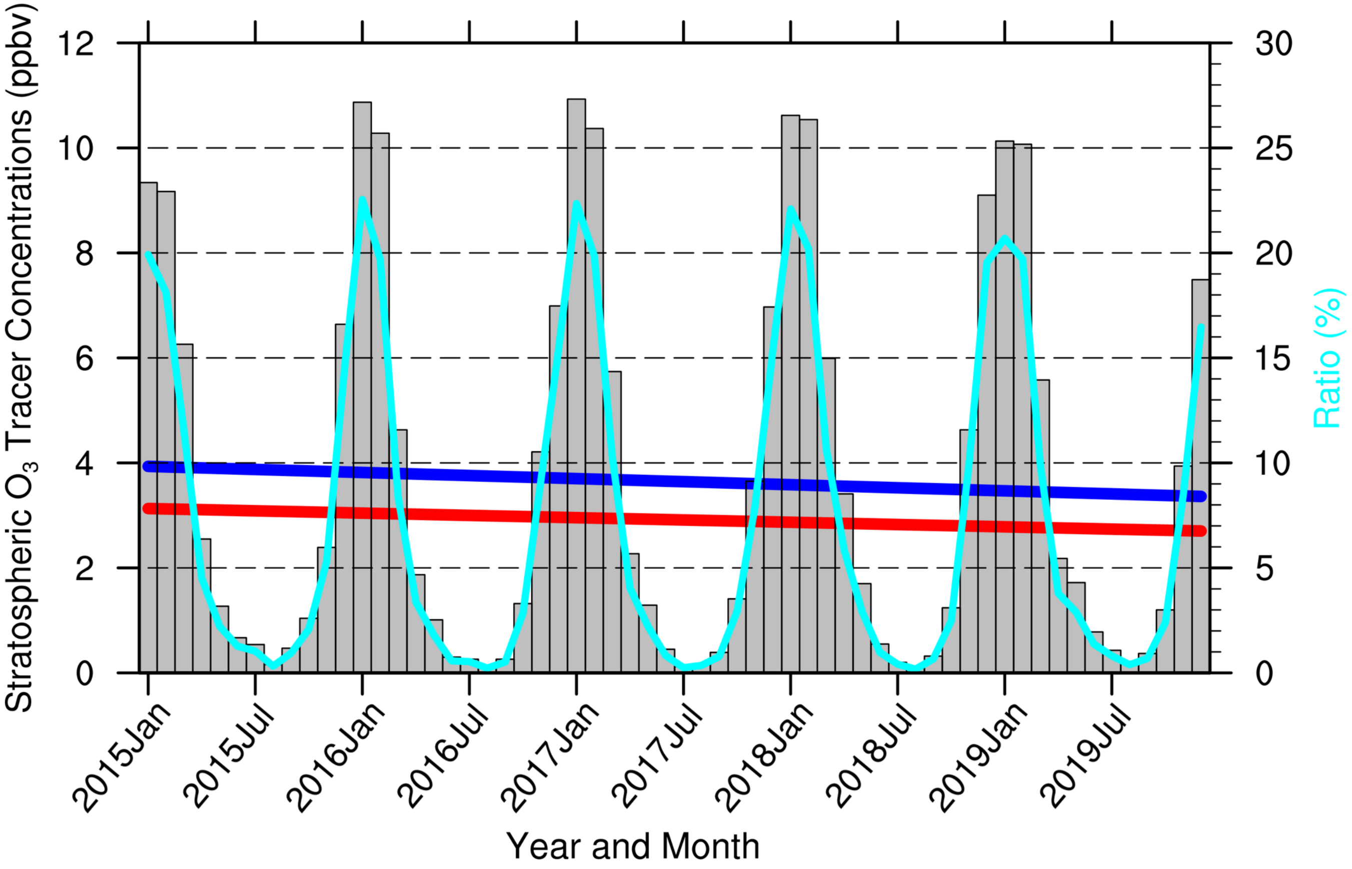

Supplementary Figure 5. Monthly stratospheric O_3 tracer concentrations from the MERRA2-GMI global atmospheric chemistry model. Stratospheric O_3 tracer at the lowest model level (grey bars, unit: ppbv) was averaged over China since January 2015 with a linear regression fit (blue line) based on the MERRA2-GMI (0.5°X0.5°) simulations. The ratios of stratospheric O_3 tracer concentrations to the overall surface O_3 concentrations and their linear regression fit are shown by the cyan line and red line, respectively.

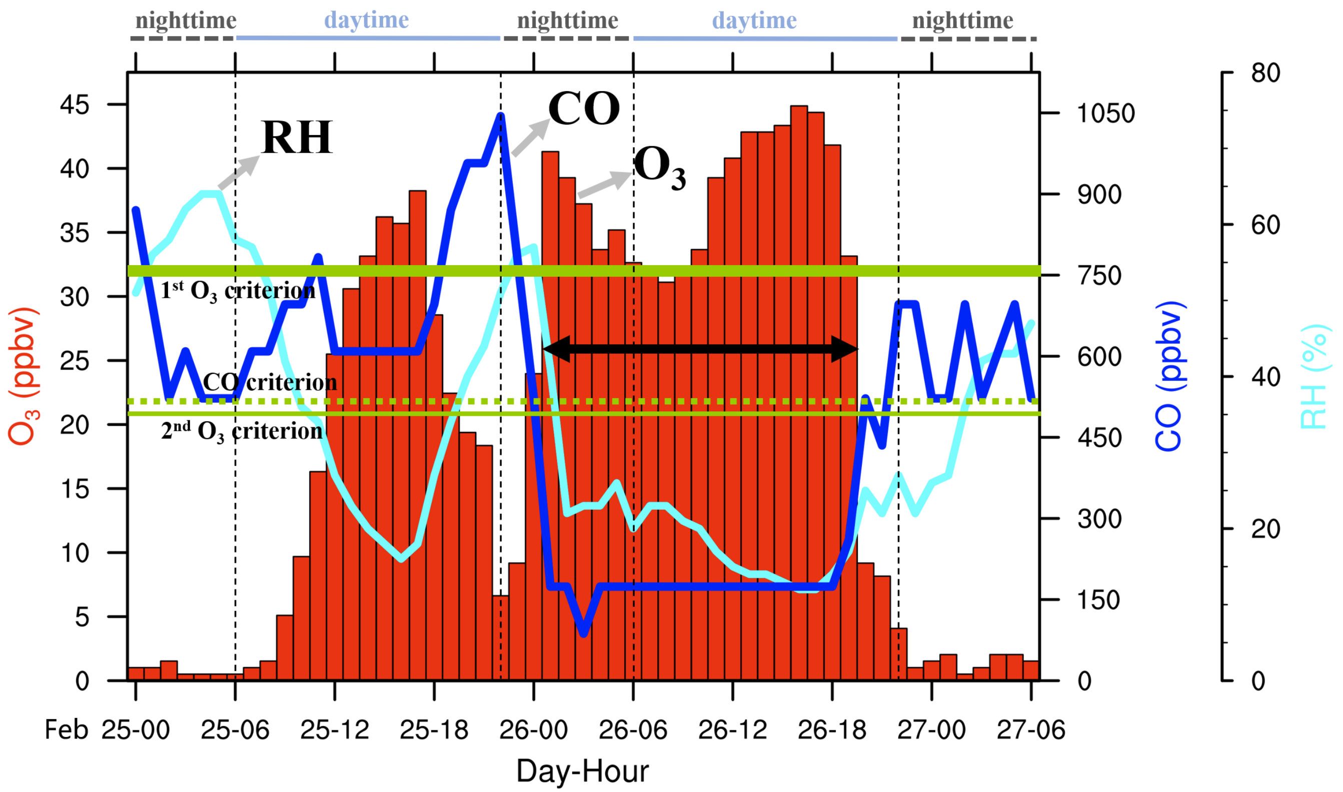

Supplementary Figure 6. Illustration of the detection of a stratospheric intrusion to the surface (SITS) event based on surface O_3 and CO observations. The red bar shows hourly O_3 concentrations (unit: ppbv) during 25-27 February 2022 in Beijing. The thin and thick solid green lines represent the mean noontime O_3 concentration in the corresponding season ($\overline{O_3}^{noon}$, 1st O_3 criterion) and the seasonal mean O_3 concentration ($\overline{O_3}^{season}$, 2nd O_3 criterion). The blue line shows the concurrent measurements of CO concentrations (unit: ppbv), and the dashed green line represents the seasonal mean of CO concentration. The horizontal black arrow represents the start and end timing of the detected SITS event (duration). This SITS event started at 01:00 (Local Time, LT) on 26 February 2020 when both the change rates of O_3 and CO as well as their absolute concentrations satisfied the predefined criteria (see Methods). The SITS ended at 19:00 LT on 26 February 2020 after which O_3 declined below the $\overline{O_3}^{season}$ value. A synchronous decline of relative humidity (RH, unit: %) occurred during the SITS event as shown by the cyan line, indicating stratospheric dryness reaching the surface.

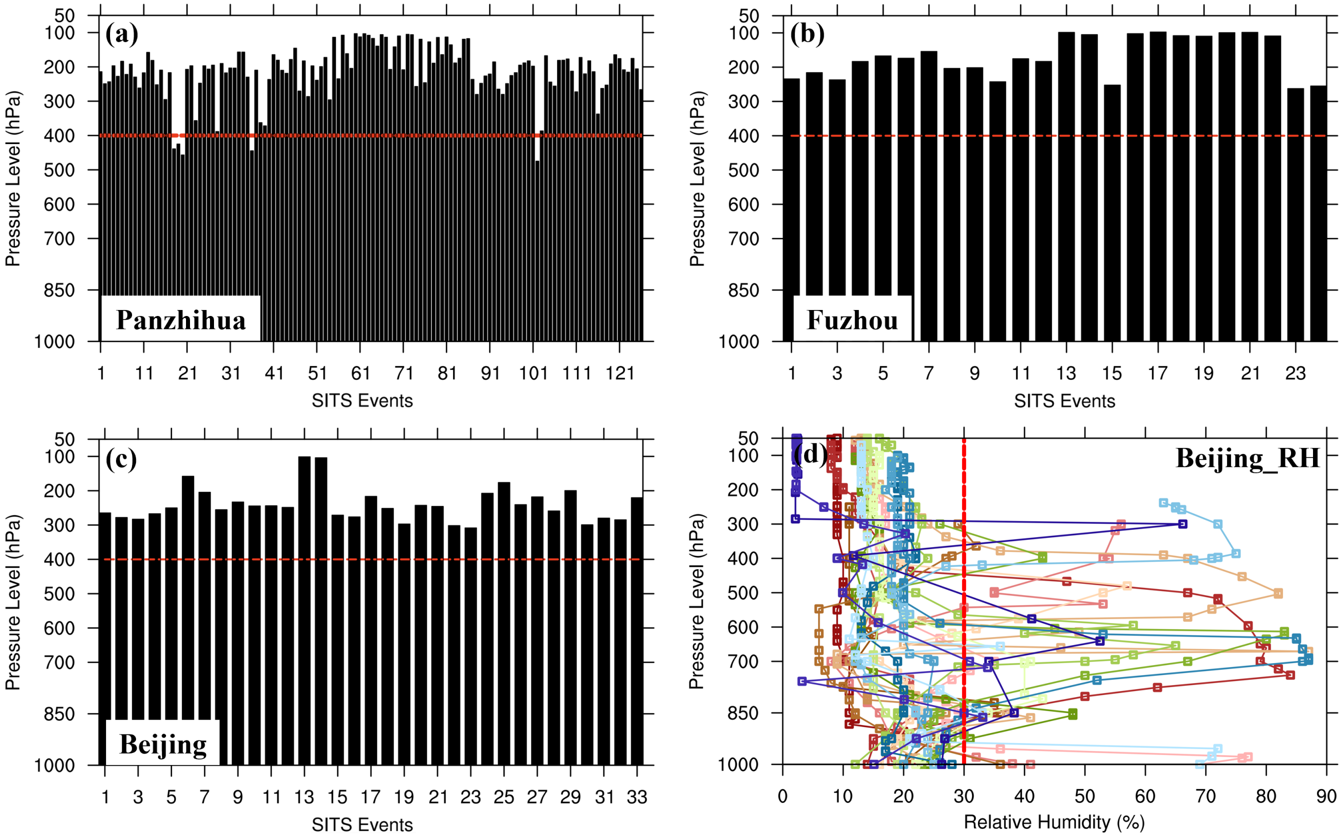

Supplementary Figure 7. The origin heights of airmass associated with stratospheric intrusions to the surface (SITS) events in selected cities, based on the HYSPLIT backward trajectory simulations. The information of the start hour for each SITS in (a) Panzhihua, (b) Fuzhou and (c) Beijing is used to initiate HYSPLIT, and the 400-hPa level is highlighted by the horizontal red dashed line. (d) Composite of relative humidity (RH, unit: %) profiles over Beijing during the 33 SITS events. The stratospheric dryness is highlighted by the vertical dashed red line (RH=30%).

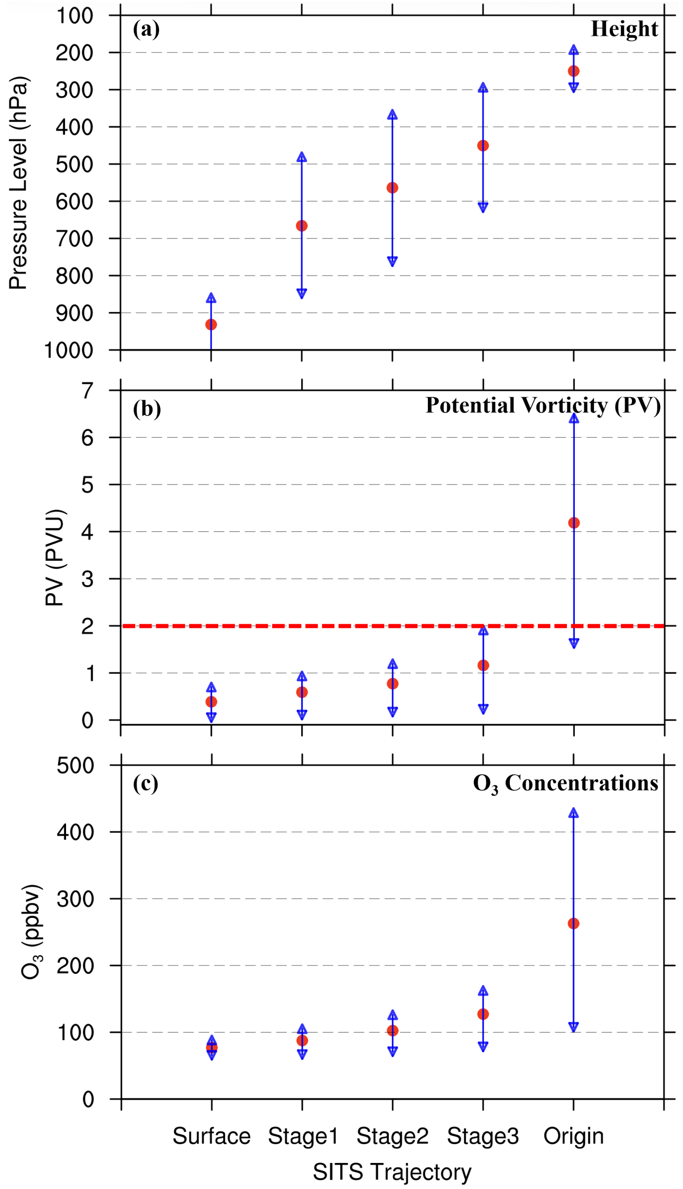

Supplementary Figure 8. Statistics of height, potential vorticity (PV) values, and O_3 concentrations during different travel stages of airmass associated with the stratospheric intrusions to the surface (SITS) events in China over 2015-2022. The information on the start hour and location for each SITS is used to initiate HYSPLIT. The maximum height of each 10-day backward trajectory is regarded as the origins. PV values and O_3 concentrations along the trajectory are extracted from MERRA-2 reanalysis data. Given the different transport routes and travel time of trajectories of all the SITS events, the travel time is evenly divided into three parts (referred to as Stage 1, Stage 2 and Stage 3). The mean height, PV values and O_3 concentrations in each stage are shown by the red dots, and the blue arrows measure the 10th and 90th percentile of these parameters. The horizontal red line (2 PVU) in Supplementary Fig. 8b indicates the iso-surface of the dynamical tropopause.
