# Supplementary figures and images for: Stratospheric influence on surface ozone pollution in China
Source: Nat Commun. 2024 May 14;15:4064. doi: 10.1038/s41467-024-48406-x (PMC11093980; doi:10.1038/s41467-024-48406-x)

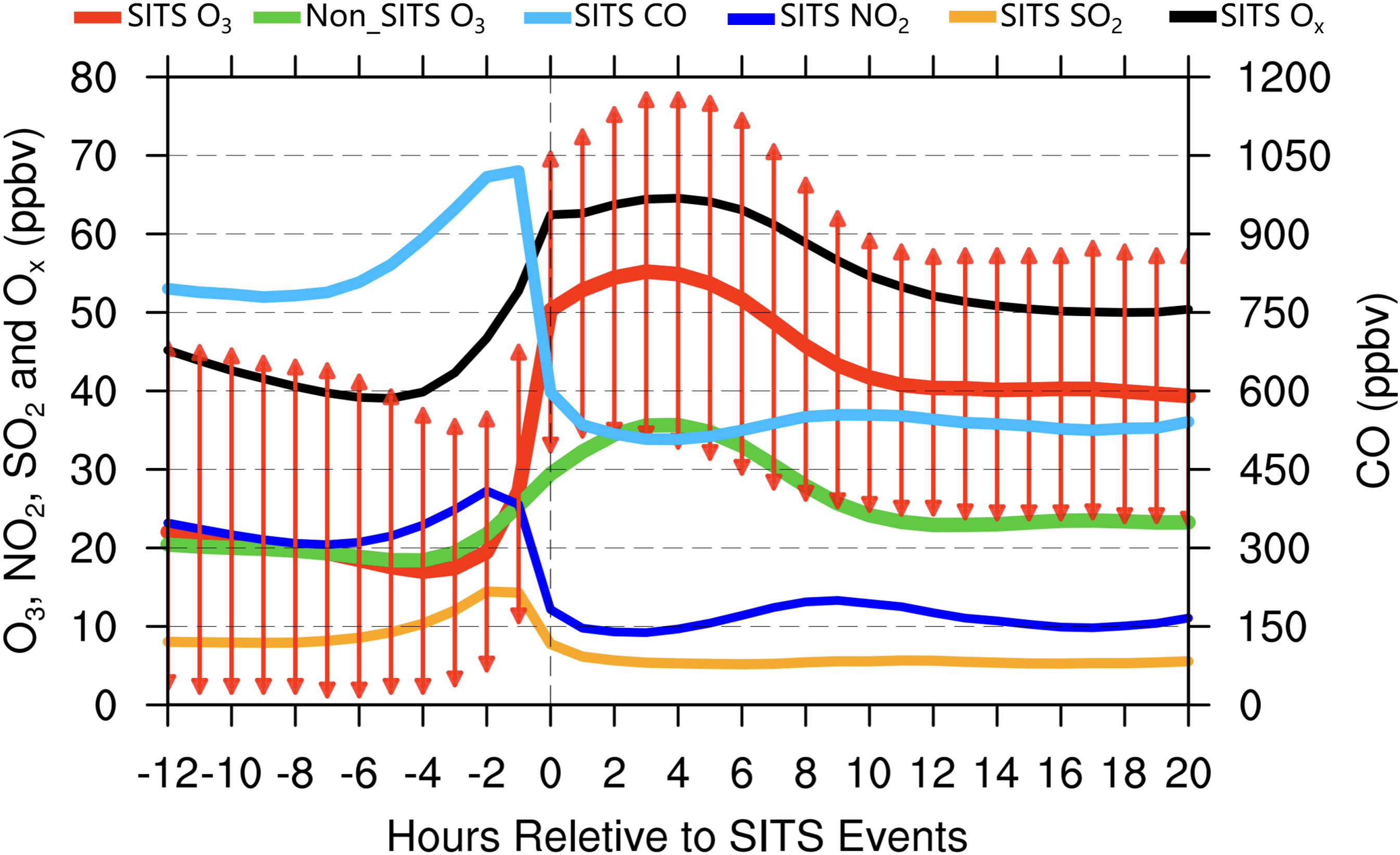

Supplement: Supplementary file 3 — Source Data [file 41467_2024_48406_MOESM3_ESM.pdf]
